# Targeting autophagy potentiates antitumor activity of Met-TKIs against Met-amplified gastric cancer

**DOI:** 10.1038/s41419-019-1314-x

**Published:** 2019-02-13

**Authors:** Xiaoting Lin, Zhi Peng, Xiaojuan Wang, Jianling Zou, Dongshao Chen, Zuhua Chen, Zhongwu Li, Bin Dong, Jing Gao, Lin Shen

**Affiliations:** 1 Department of Gastrointestinal Oncology, Peking University Cancer Hospital & Institute, Beijing, 100142 China; 2 Department of Pathology, Key laboratory of Carcinogenesis and Translational Research (Ministry of Education/Beijing), Peking University Cancer Hospital & Institute, Beijing, 100142 China

## Abstract

Met tyrosine kinase inhibitors (Met-TKIs) subjected to ongoing clinical trials are a promising option for Met-amplified gastric cancer (GC), but how to optimize their antitumor activity especially with combination schemes remains unclear. Since autophagy is known to be initiated by Met-TKIs, we investigated its underlying mechanisms and therapeutic potentials of Met-TKIs combined with autophagy inhibitors against Met-amplified GC. As expected, four Met-TKIs induced autophagy in Met-amplified GC cells marked by p62 degradation, LC3-II accumulation and increased LC3-positive puncta. Autophagy flux activation by Met-TKIs was further validated with combined lysosomal inhibitors, bafilomycin A1 (Baf A1) and hydroxychloroquine (HCQ). Molecular investigations reveal that autophagy induction along with mTOR and ULK1 de-phosphorylation upon Met-TKI treatment could be relieved by hepatocyte growth factor (HGF) and mTOR agonist MHY1485 (MHY), suggesting that autophagy was initiated by Met-TKIs via Met/mTOR/ULK1 cascade. Intriguingly, Met-TKIs further suppressed cell survival and tumor growth in the presence of autophagy blockade in Met-amplified GC preclinical models. Thus, these findings indicate Met/mTOR/ULK1 cascade responsible for Met-TKI-mediated autophagy and Met-TKIs combined with autophagy inhibitors as a promising choice to treat Met-amplified GC.

## Introduction

Despite recent improvements in anticancer therapeutics, clinically available drugs for gastric cancer (GC) are limited, and hence GC remains a leading cause of mortality in China^1^. Receptor tyrosine kinase Met (also known as hepatocyte growth factor (HGF) receptor) is a promising target for Met-addicted GC. The HGF/Met pathway broadly participates in GC survival, invasion and metastasis^2^, and aberrant activation of HGF/Met pathway represented by Met overexpression and gene amplification frequently occurs in GC^3^. Met overexpression and amplification were found in 39% and 7% of advanced GC in our previous study, respectively^4^. Growing evidence suggests Met gene amplification rather than protein overexpression as a true oncogenic driver and a predictive marker for Met-TKIs in GC^5–7^. Several Met tyrosine kinase inhibitors (Met-TKIs) including crizotinib (Criz) and volitinib (Voli) against Met-amplified GC are being investigated for this reason.

Targeted drugs usually elicit better antitumor activity when combined with chemotherapy or other inhibitors due to known or unknown mechanisms^8^. Therefore, understanding signaling pathway changes resulting from Met-TKI treatment are very critical to develop novel combination strategies for improving Met-TKI efficacy, especially in the Met-amplified subpopulation. Based on accumulating data, autophagy is frequently induced by drug exposure and acts as an attractive molecular target to potentiate efficacy of anticancer treatment^9–19^. Autophagy, a cellular adaptive response to stresses including anticancer agents, is an evolutionally conserved proteolytic process involving lysosomal degradation and recycling damaged cellular components and energy to maintain homeostasis^20^. Of note, protective autophagy rising in most contexts poses an opportunity for autophagy inhibitor-based combination therapies. Autophagy blockade has been applied concurrently with either chemotherapies or targeted therapies to optimize their efficacy in various cancers in preclinical studies^9,10,12–16,19,21,22^. Regarding GC, a previous study roughly revealed that targeting autophagy initiated by Met-TKIs improved Met-TKI efficacy in vitro^23^; however, Met-TKI-associated autophagy flux alterations, mechanisms underlying autophagy induced by Met-TKIs and therapeutic potentials of dual targeting Met/autophagy in Met-amplified GC, especially in vivo, remain far from clear. Hence, this study aims at these issues to deepen our understanding of potentials of optimizing Met-TKI efficiency with targeting autophagy in Met-addicted GC.

## Results

### Met-TKIs induced autophagy in Met-amplified GC cells

**Fig. 1:**
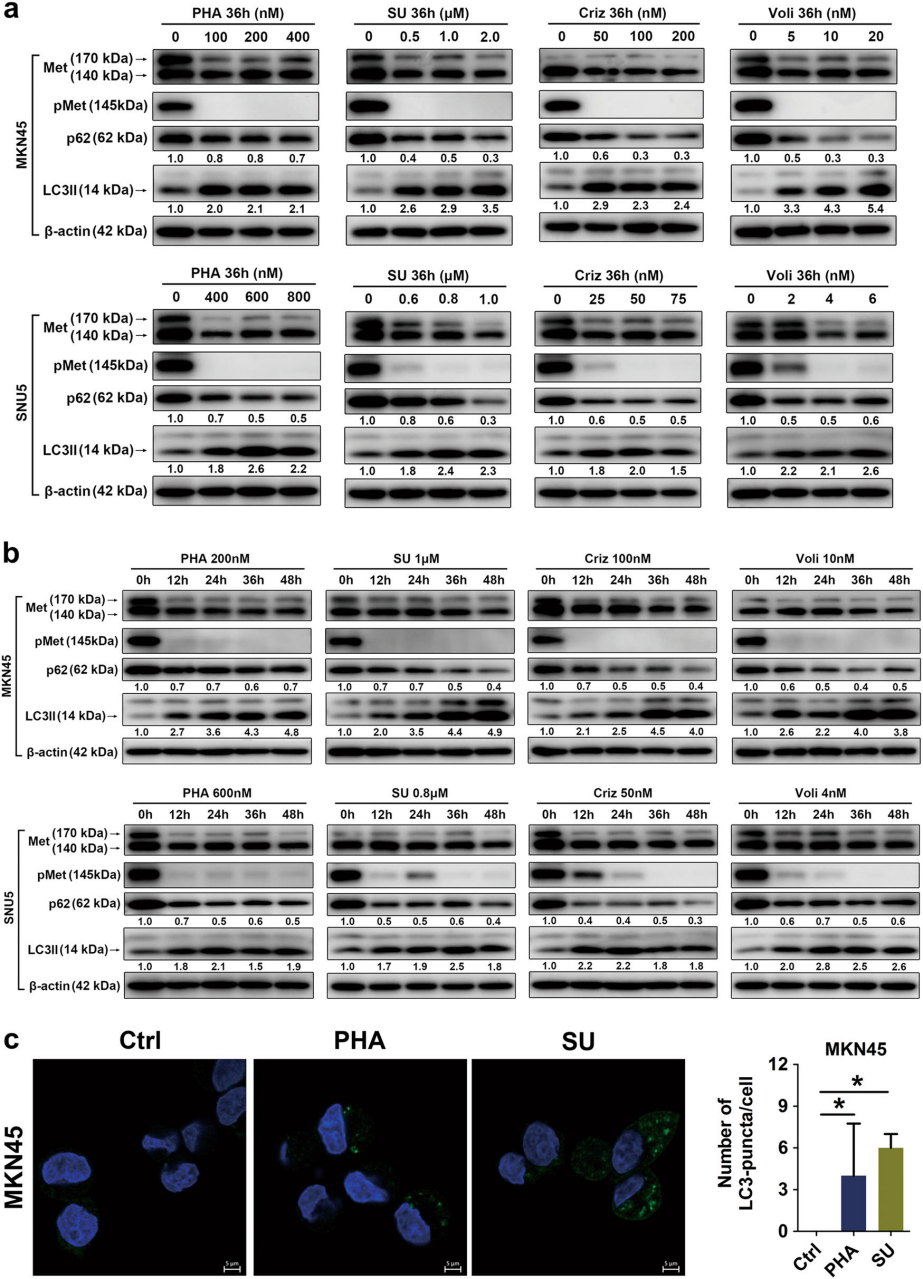
Met tyrosine kinase inhibitors (Met-TKIs) induced autophagy in Met-amplified gastric cancer (GC) cells. **a**, **b** Met-amplified GC cells were treated with Met-TKIs as indicated and lysates were immunoblotted for proteins. **c** MKN45 cells were treated with PHA-665752 (PHA) 200 nM or SU11274 (SU) 1 μM for 36 h and LC3-positive puncta were counted by confocal immunofluorescence (autophagosomes were green and 4′,6-diamidino-2-phenylindole (DAPI)-stained nuclei were blue). Scale bar, 5 μm. Data are expressed as median with interquartile range. *P < 0.05 by nonparametric Mann–Whitney *U*-test

**Fig. 2:**
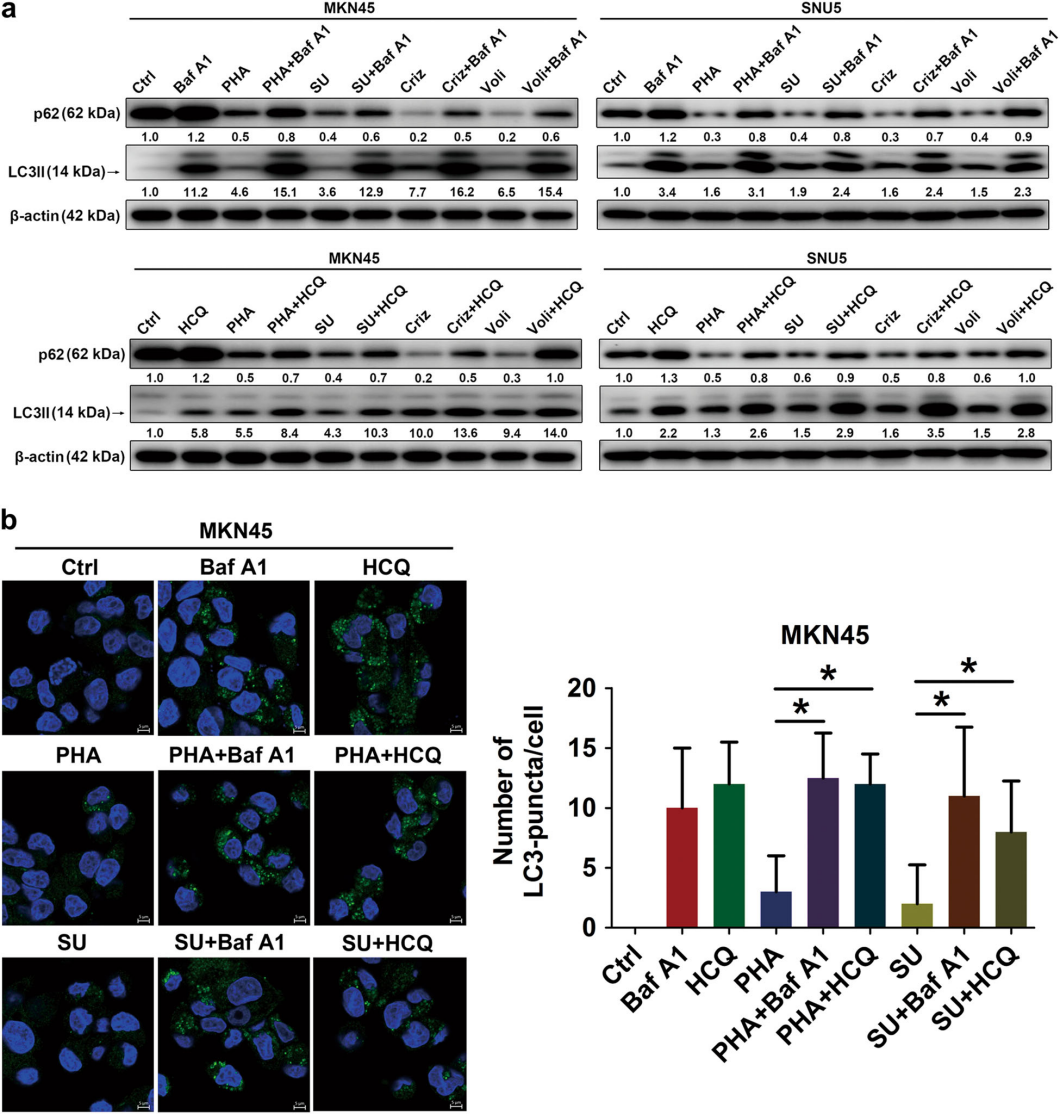
Met tyrosine kinase inhibitors (Met-TKIs) activated autophagy flux in Met-amplified gastric cancer (GC) cells. **a**, **b** After exposure to Met-TKIs (PHA-665752 (PHA) 200/600 nM, SU11274 (SU) 1/0.8 μM, crizotinib (Criz) 100/50 nM or volitinib (Voli) 10/4 nM for MKN45 and SNU5 cells, respectively) with/without autophagy inhibitors (bafilomycin A1 (Baf A1) 10 nM or hydroxychloroquine (HCQ) 10 μM) for 36 h, cell lysates were immunoblotted for indicated proteins and LC3-positive puncta were counted by confocal immunofluorescence (autophagosomes were green and 4′,6-diamidino-2-phenylindole (DAPI)-stained nuclei were blue). Scale bar, 5 μm. Data are expressed as median with interquartile range. *P < 0.05 by nonparametric Mann–Whitney *U*-test

Met-amplified GC cells^6,24,25^ were treated with various doses and duration of Met-TKIs. Met-TKIs actioned on Met-amplified GC cells, indicated by remarkable de-phosphorylation of Met (Fig. 1a, b). Of note, the total Met levels, both its pro-Met and Met form, tended to be reduced by Met-TKIs to some degree. Marked by degradation of p62 and accumulation of LC3-II, autophagy was initiated after Met-TKI treatment (Fig. 1a, b). LC3-positive puncta consistently increased compared to the control group (Fig. 1c). Thus, Met-TKIs induced autophagy in Met-amplified GC cells. As reported, LC3-II accumulates due to either increased autophagy flux or decreased autophagy degradation, which can be distinguished with combined lysosomal inhibitors^26^. Degradation of p62 in Met-amplified GC cells exposed to Met-TKIs was blocked while LC3-II accumulation and LC3-positive puncta increased in the presence of lysosomal inhibitors (bafilomycin A1 (Baf A1) and hydroxychloroquine (HCQ); Fig. 2a, b). These data suggest that autophagosome formation rather than blockade of autophagy degradation occurred upon Met-TKI treatment. Hence, Met-TKIs activated autophagy flux in Met-amplified GC cells.

### Met-TKIs induced autophagy via Met/mTOR/ULK1 signaling pathway

**Fig. 3:**
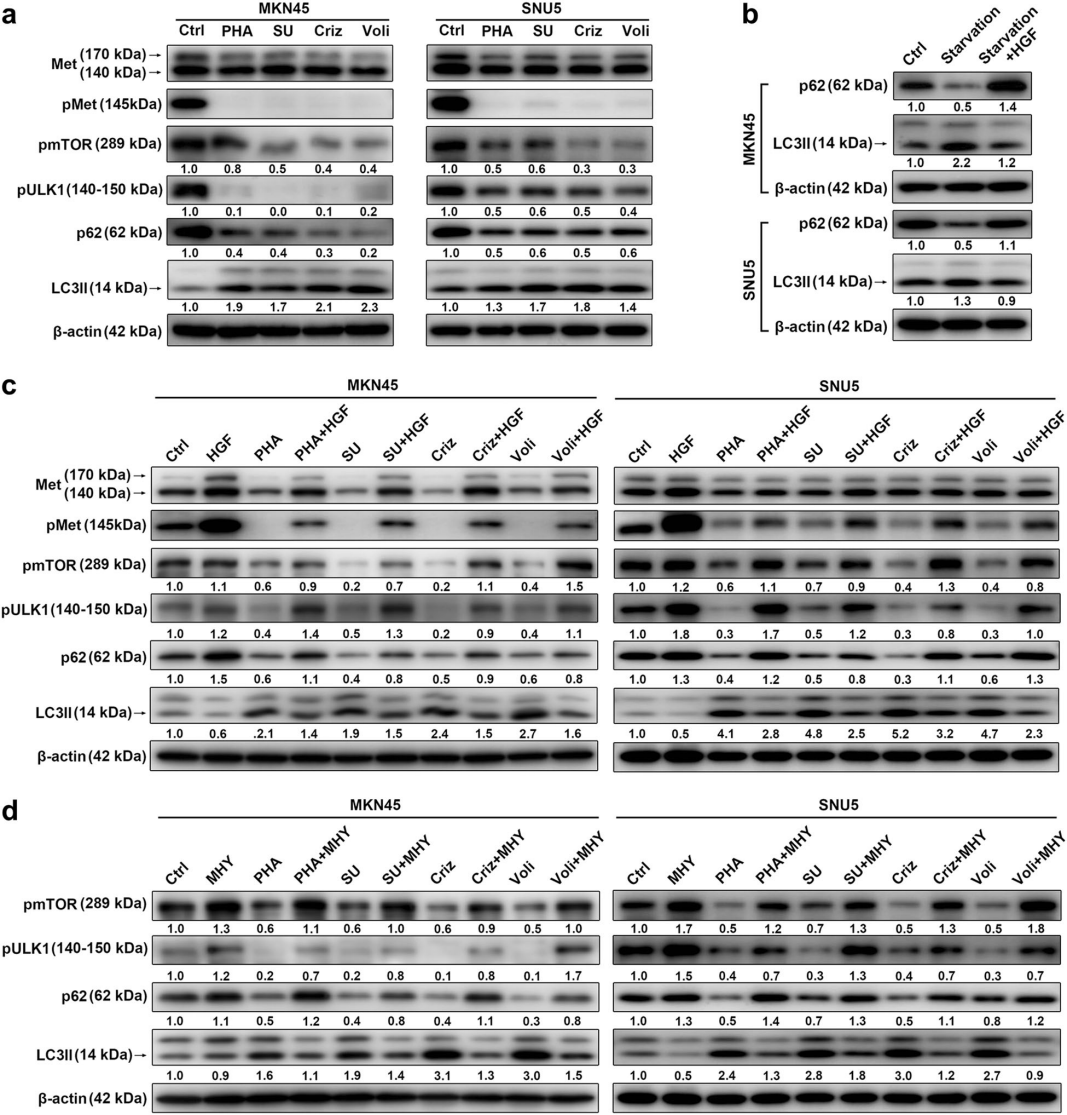
Met tyrosine kinase inhibitors (Met-TKIs) induced autophagy via Met/mTOR/ULK1 signaling pathway. **a**–**d** Gastric cancer (GC) cell lysates were immunoblotted for indicated proteins after exposure to Met-TKIs (PHA-665752 (PHA) 200/600 nM, SU11274 (SU) 1/0.8 μM, crizotinib (Criz) 100/50 nM or volitinib (Voli) 10/4 nM for MKN45 and SNU5 cells, respectively) or starvation by serum-free medium with/without hepatocyte growth factor (HGF; 50 ng/ml for 30 min) or mammalian target of rapamycin (mTOR) agonist MHY1485 (MHY; 5 μM) for 36 h

To excavate potential molecular mechanisms, we next evaluated phosphorylation status of mTOR (mammalian target of rapamycin, a common downstream target of HGF/Met signaling and core autophagy pathway) and its autophagy downstream effector ULK1^26^. Met, mTOR, and ULK1 were de-phosphorylated when Met-TKIs induced autophagy, suggesting that mTOR inactivation and ULK1 activation were responsible for autophagy initiation by Met-TKIs (Fig. 3a). As a ligand of Met, HGF can be used as a pMet activator, evidenced by reducing Met-TKI-caused Met de-phosphorylation (Fig. 3c). Of interest, HGF not only inhibited autophagy mediated by starvation (Fig. 3b) but also attenuated autophagy along with mTOR and ULK1 de-phosphorylation initiated by Met-TKIs (Fig. 3c). Meanwhile, these Met-TKI actions could also be diminished by an mTOR agonist MHY1485 (MHY) (Fig. 3d). Taken together, Met-TKIs induced autophagy via Met/mTOR/ULK1 cascade in Met-amplified GC cells.

### Autophagy blockade enhanced antitumor activity of Met-TKIs ex vivo and in vivo

**Fig. 4:**
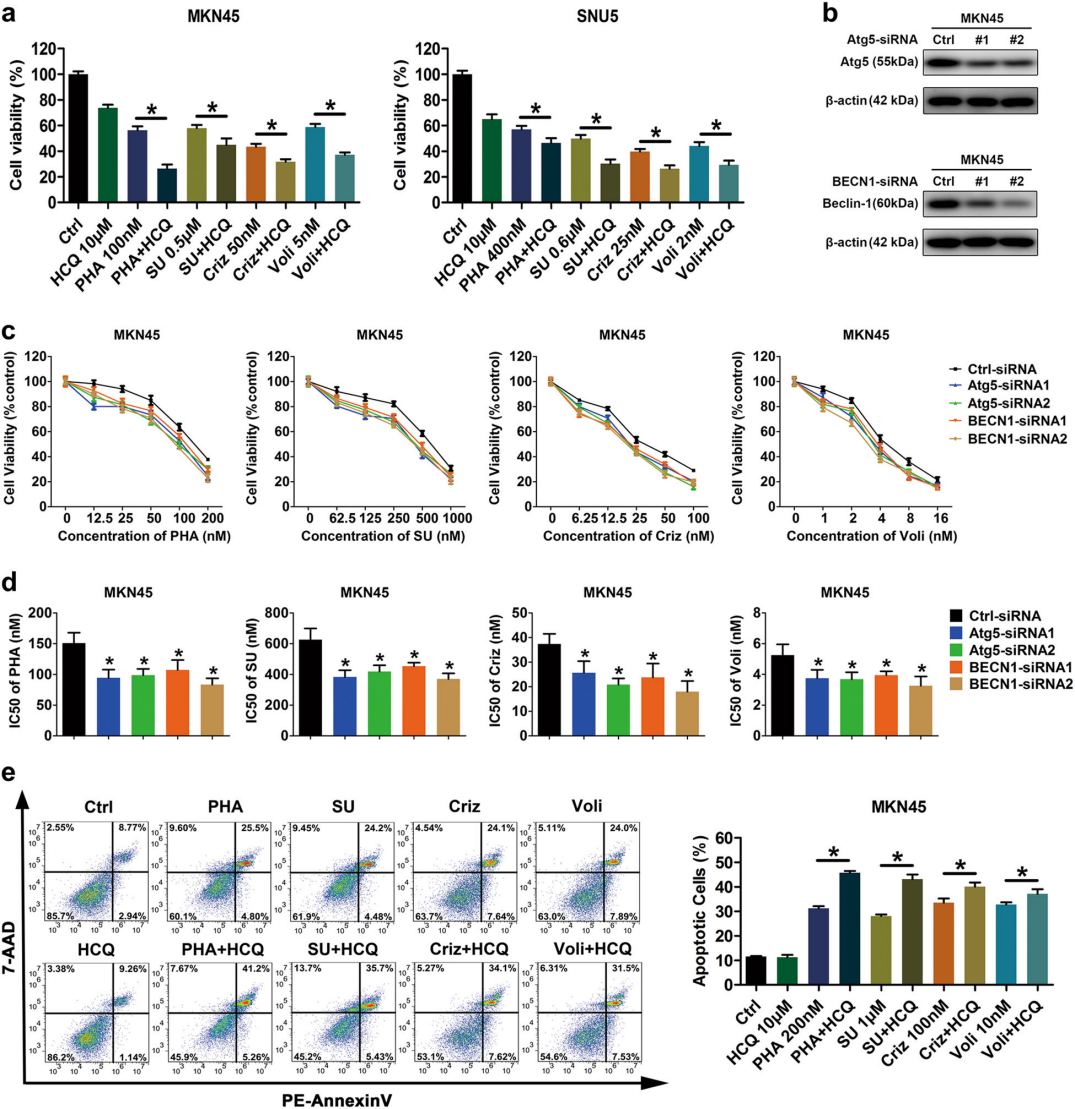
Autophagy blockade enhanced antitumor activity of Met tyrosine kinase inhibitors (Met-TKIs) ex vivo. **a** Cell viability was measured by CCK-8 assay in Met-amplified gastric cancer (GC) cells exposed to Met-TKIs with/without the autophagy inhibitor hydroxychloroquine (HCQ) for 48 h. Data were expressed as Mean ± SD. *P < 0.05 by one-way analysis of variance (ANOVA). **b** Following Atg5 and BECN1 knockdown by small interfering RNA (siRNA), immunoblots for Atg5 and Beclin1 were performed. **c** Sensitivity of Atg5-knockdown or BECN1-knockdown MKN45 cells and their controls to Met-TKIs were compared using CCK-8 assay. **d** The half-maximal inhibitory concentrations (IC_50_) were presented as mean ± SD of three independent experiments. *P < 0.05 by two-tailed Student’s *t*-test. **e** Apoptosis analysis using Annexin V-phycoerythrin (PE)/7-amino-actinomycin (7-AAD) double staining was carried out in MKN45 cells exposed to Met-TKIs for 36 h with/without the autophagy inhibitor HCQ for 12 h. Data were expressed as mean ± SD. *P < 0.05 by one-way ANOVA

**Fig. 5:**
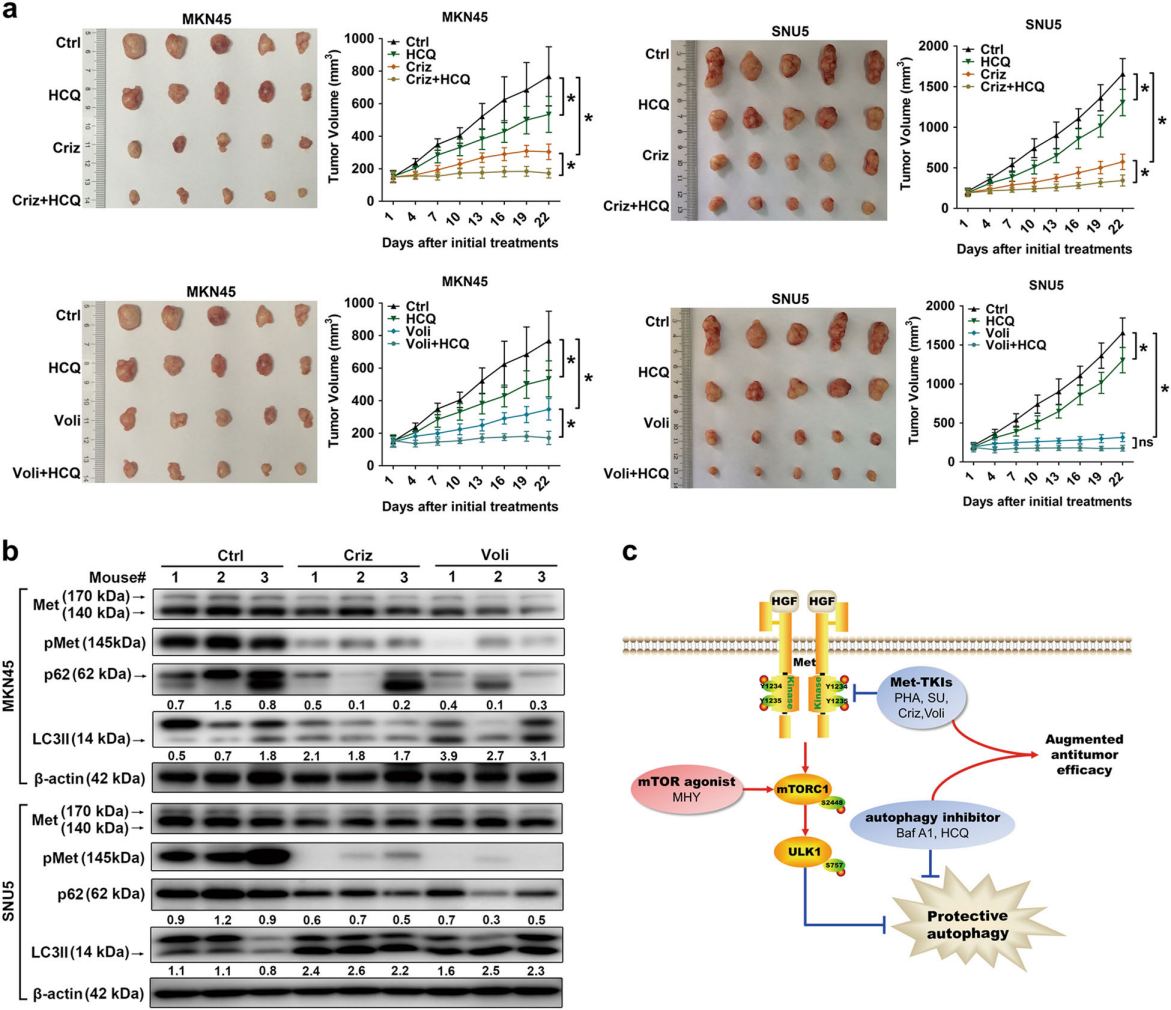
Autophagy inhibitor hydroxychloroquine (HCQ) enhanced antitumor activity of Met tyrosine kinase inhibitors (Met-TKIs) in vivo. **a** Crizotinib (Criz; 12.5 mg/kg/day, by gavage) and volitinib (Voli (5 mg/kg/day, by gavage) with/without HCQ (60 mg/kg/day, intraperitoneally (i.p.)) were given to mice bearing gastric cancer (GC) tumors for 21 days. Tumor measurements were performed every 3 days after treatment and xenograft growth curves were presented. Data were expressed as mean ± SD. *P < 0.05 for last measurement by two-way analysis of variance (ANOVA) with post hoc Bonferroni correction; ns, not significant. **b** Tumor lysates were immunoblotted for autophagy proteins. **c** Representation of augmented antitumor effects of dual Met/autophagy blockade compared with Met-TKIs alone

Prevalently, autophagy is considered as a pro-survival mechanism in many cancers^20^. Given Met-TKI-mediated autophagy presented in our Met-amplified GC cells, dual inhibition of Met and autophagy was proposed. Compared to controls, Met-TKIs or the autophagy inhibitor HCQ alone inhibited cell viability to some extent, while the combination groups had a better response in Met-amplified GC cells (Fig. 4a). Given that HCQ can exert cytotoxicity independent of autophagy^26^, targeting autophagy by small interfering RNA (siRNA) interference was employed to further confirm augmented effects of autophagy blockade on Met-TKI cellular lethality. As anticipated, both Atg5 and BECN1 knockdown could sensitize the Met-amplified MKN45 cell to Met-TKIs (Fig. 4b–d). Due to cross-talks between autophagy and cell apoptosis^27^, we also detected the combination’s effect on apoptosis. As revealed, Met-TKI-mediated apoptosis increased in the presence of HCQ in terms of total apoptotic cells (Fig. 4e). However, the phenomena could arise in the context of increased necrotic cells by HCQ non-specific cytotoxicity and early-phase apoptotic cells were almost unaffected, which indicated that apoptotic cell death might not as necessarily as growth inhibition linked to strengthened cytotoxicity of Met-TKIs plus autophagy inhibitors in Met-amplified GC cells. Paralleled to the in vitro findings, clinically investigated Met-TKIs (Criz and Voli) or the autophagy inhibitor HCQ alone reduced tumor growth, while Met-TKIs combined with HCQ yielded the greatest tumor suppression in Met-amplified GC xenografts (Fig. 5a). Of note, the combination of Voli and HCQ did not reach the level of statistical significance compared to Voli monotherapy in mice bearing SNU5 cells. Besides, autophagy occurred in tumors treated with Met-TKIs as indicated by p62 degradation and LC3-II accumulation (Fig. 5b). These data suggest that Met-TKIs elicited greater antitumor activity in the presence of autophagy blockade in Met-amplified GC preclinical models.

## Discussion

Continuous efforts to optimize Met-targeted therapeutic strategies led to the identification of Met amplification as a possible driver event of GC^5,7^, which fosters clinical trials on Met-TKI therapeutic potentials against Met-amplified GC (www.clinicaltrials.gov). However, efficacy of Met-TKIs alone is limited in Met-addicted GC. Different approaches represented by combination designs, therefore, are being studied to improve overall response.

To develop feasible drug combination strategies, molecular or signaling pathway changes after treatment must be known. Chemotherapies and targeted therapies have been validated to induce autophagy in solid tumors and in hematologic malignancies^9–19,28–30^. Protective autophagy frequently occurs after drug stimulation, indicative of potential benefits with combined autophagy inhibitors. Based on published results^17,23,31^, our data further display autophagy induction after exposure to Met-TKIs in Met-amplified GC preclinical models (Figs. 1 and 5b), with treatment dose and duration took into consideration. Of note, autophagy in the in vivo scenario will be strengthened by other evidence like HCQ-mediated LC3  accumulation^32,33^ and immunohistochemistry analysis of autophagy-associated proteins^26^. Using lysosomal inhibitors recommended in a guideline^26^, autophagy flux activation rather than repressed autophagy degradation by Met-TKIs was further elucidated (Fig. 2), which enabled autophagy modulators to be added in the treatment of Met-amplified GC. Of note, lysosomal inhibitors (Baf A1 and HCQ) only partially reversed p62 reduction induced by Met-TKIs (Fig. 2a). To further confirm decreased p62 levels dependent on autophagy, neighbor of BCRA1 gene 1 (NBR1) that shares a similar function with p62 in autophagic degradation^26,34^ was used. As expected, Met-TKIs downregulated NBR1 expressions in Met-amplified GC cells, which could be mitigated by lysosomal inhibitors (Fig. S2). However, these results did not exclude the possibility of p62 reduction by Met-TKIs through alternative mechanisms such as proteasomal degradation and caspase-induced cleavage^26^, which remains to be deciphered using proteasome and pan-caspase inhibitors. Meanwhile, other assays for monitoring autophagy such as transmission electron microscopy and immunofluorescence experiments can be introduced since confocal findings were less clear (Figs. 1c and 2b), especially for Met-TKI monotherapy groups. Besides, whether autophagy occurs in Met non-amplified GC cells is unclear. Despite limited effects on cell proliferation compared to Met-amplified GC cells, Met-TKIs initiated autophagy in Met non-amplified NCI-N87 cells (Fig. S1)^6,24,25^, whose values in the treatment of GC remains elusive. In order to optimize responses of Met-addicted GC to Met-TKIs, only Met-amplified GC cells were used in this study.

Molecular mechanisms underlying Met-TKI-induced autophagy in GC are unclear. De-phosphorylation of mTOR and ULK1 (activated and inactivated site, respectively) involves in autophagy^26,35^ and was observed upon Met-TKI treatment in Met-amplified GC cells (Fig. 3a). Met/mTOR/ULK1 cascade responsible for Met-TKI-mediated autophagy was thus further verified by combined HGF (pMet activator) and MHY (mTOR agonist), marked by antagonizing Met-TKI actions on autophagy, mTOR, and ULK1 de-phosphorylation (Fig. 3c, d). These phenomena consistently occur in cardiac disease and hepatocellular carcinoma^35,36^. However, the molecular events following ULK1 de-phosphorylation and signal transducer and activator of transcription 3 (STAT3) pathway’s role during the process as reported in lung cancer^17^ merit future investigations.

Autophagy after antitumor treatment may reduce efficacy of therapeutics^9–17,19^, in accordance with potentiated anticancer activity of Met-TKIs plus autophagy inhibition against Met-amplified GC preclinical models (Figs. 4 and 5a, c). These data suggest therapeutic potentials of the combination strategy in Met-amplified GC, which can be warranted by assessing impacts of this combination scheme on other events involving both HGF/Met pathways and autophagy, such as cancer progression, angiogenesis, and antitumor immune response^18,37–39^. Of note, the superiority of Voli plus HCQ over Voli alone was presented in mice bearing SNU5 cells without statistical significance (Fig. 5a), which may result from striking efficacy of Voli monotherapy. On the other hand, available autophagy inhibitors can exert non-specific cytotoxicity, targeting autophagy to improve Met-TKI efficacy was further proven by knockdown of autophagy-related molecules (Atg5 and BECN1; Fig. 4b–d), and novel more specific or potent autophagy inhibitors like Lys05 and SAR405^40,41^ are expected to promote basic studies and enter clinical practice. Moreover, apoptotic cell death contributed to augmented efficacy of this combination strategy in terms of total apoptotic cells (necrotic cells included) but early-phase apoptotic cells were almost unaffected (Fig. 4e), inconsistent with a previous finding that autophagy inhibitor CQ mitigated Criz-induced apoptosis in Met-amplified GC cells^31^. This discrepancy might be explained by different treatment doses and durations of autophagy inhibitors, reflecting a dynamic process of autophagy and non-specific cytotoxicity of autophagy inhibitors to some extent. In line with the complexity, whether autophagy inhibited or enhanced apoptosis mediated by anticancer agents is controversial in different studies due to a variety of confounding factors^10,12,13,19,21,28–30,35,42^.

Xenografts derived from Met-amplified GC cells were used for in vivo experiments but data would be more compelling if patient-derived xenograft (PDX) models were applied. Met amplification occurred in about 7% of patients with advanced GC in our retrospective study^4^ while merely about 1–2% in our ongoing prospective clinical trial (NCT01985555). Similar to the low Met amplification rate, none of our established 50 GC PDXs featured Met amplification^43^. Efficacy of Met-TKIs plus autophagy inhibitors can be further verified when Met-amplified PDXs are available. On the other hand, our previous studies revealed a pMet-positive rate of 6% in patients with advanced GC, a strong association between pMet expression and Met amplification, and Voli-elicited strong antitumor activity in a GC PDX model with pMet expression^4,43^. Indeed, GC cells (MKN45 and SNU5) were pMet positive and Met-TKI-based treatment obviously inhibited Met phosphorylation in this work (Figs. 1 and 3 and 5b). Thus, patients with Met amplification/pMet expression may be suitable for Met-TKI combined with autophagy inhibitors. Moreover, we notice that Met-TKIs caused Met de-phosphorylation accompanied by reduced expressions of pro-Met and Met (Figs. 1, 3 and 5b), indicating total Met protein levels affected by Met-TKIs. However, total Met protein levels can be decreased, unaffected, or increased when Met-TKIs yield anticancer activity and markedly inhibit Met phosphorylation^24,25,44,45^. Met phosphorylation status, thus, outweighs total Met protein levels for Met-TKI antitumor actions, consistent with Met-TKI pharmacological mechanism. The significance of total Met expressions modified by Met-TKIs remains unrevealed.

### Conclusions

Met-TKIs induced protective autophagy via Met/mTOR/ULK1 cascade and Met-TKIs combined with autophagy inhibitors augmented antitumor activity in Met-amplified GC preclinical models, shedding light upon this combination strategy in the treatment of Met-amplified GC.

## Materials and methods

### Reagents and antibodies

PHA-665752 (PHA), SU11274 (SU), Criz, Baf A1, HCQ, and MHY were purchased from Selleck Chemicals (Houston, TX). Voli was kindly provided by AstraZeneca (Cambridge, UK). HGF was purchased from Life Technologies (Frederick, MD). Reagents were formulated and stored following the manufacturer’s protocols for in vitro and in vivo experiments. Voli was formulated in a 0.5% (v/v) carboxymethylcellulose sodium solution for mice^6^. Primary antibodies against Met, pMet (Y1234/1235), p62, LC3, mTOR, pmTOR (S2448), pULK1 (S757), Atg5, Beclin-1, NBR1, and secondary horseradish peroxidase-conjugated goat anti-rabbit and anti-mouse antibodies were purchased from Cell Signal Technology (CST, Danvers, MA). Anti-β-actin primary antibody was purchased from Sigma-Aldrich (St. Louis, MO).

### Cell lines and cell cultures

GC cell lines, MKN45 and NCI-N87, were provided by Professor Youyong Lv (Peking University Cancer Hospital and Institute), while SNU5 was obtained from Hutchison Medi Pharma (Shanghai, China). MKN45 and NCI-N87 cells were cultured in RPMI-1640 medium (Gibco BRL, Gaithersburg, MD) supplemented with 10% fetal bovine serum (Gibco BRL) and 1% penicillin and streptomycin (HyClone, Logan, UT), while SNU5 cells were cultured in Iscove's modified Dulbecco's medium (Gibco BRL) replenished with 20% fetal bovine serum and 1% penicillin and streptomycin. Cells were incubated in a humidified incubator (37 °C) with 5% CO_2_.

### Western blot

Cells were lysed using a CytoBuster protein extraction reagent (Merck Millipore, Darmstadt, Germany) in the presence of protease and phosphatase inhibitor cocktail tablets (Roche, Basel, Switzerland). Protein concentration was measured by a BCA Protein Assay Kit (Beyotime, Jiangsu, China). Soluble lysates were subjected to sodium dodecyl sulfate–polyacrylamide gel electrophoresis and transferred to a polyvinylidene fluoride membrane (Merck Millipore). After blocking with 5% bull serum albumin (Amresco, Solon, OH) or fat-free milk, membranes were probed with primary antibodies at 4 °C overnight and secondary antibodies at room temperature for 1 h. Signals were visualized using Amersham Imager 600 (GE Healthcare, Chicago, IL) after incubation with Clarity Western ECL substrate (Bio-Rad, Hercules, CA). Protein expressions were quantified using ImageJ Version 1.48 software and normalized to β-actin level followed by calculations of relative ratios to controls.

### Confocal immunofluoresent staining

Cells (75,000 cells/ml) were planted into a 35 mm glass bottom dish (NEST, Jiangsu, China) and incubated overnight in complete medium. After drug treatment, cells were fixed with 4% paraformaldehyde (Solarbio, Beijing, China) for 10 min, permeabilized with 0.5% Triton X-100 (Amresco) for 20 min, and blocked with normal goat serum (ZSJB-BIO, Beijing, China) for 30 min at room temperature. Cells were probed with primary antibody against LC3B (CST, 1:200) at 4 °C overnight and Alexa Fluor 488-conjugated goat anti-rabbit IgG (Molecular Probes, Eugene, OR, 1:500) in the dark for 1 h at room temperature, whereas nuclei were counterstained with 4′,6-diamidino-2-phenylindole (DAPI; Beyotime, 1:3000) in the dark for 5 min at 4 °C. All reagents were diluted in phosphate-buffered saline (PBS) and all steps were followed by PBS-washing for three times. Images were captured with the ZEN version 2012 software (Zeiss, Gottingen, Germany) using a laser scanning confocal microscope LSM 780 (Zeiss). Dots of specific green signals within cells were considered to be LC3-positive puncta.

### Cell viability assay

Cells were seeded at a density of 4000 cells/well in 96-well plates and incubated overnight in complete medium. After drug treatment, cell viability was measured using a CCK-8 kit (Dojindo Laboratories, Tokyo, Japan) according to the manufacturer’s protocol. Absorbance was measured at 450 nm using a spectrophotometer.

### Genetic knockdown by siRNA

Atg5 and BECN1 siRNA kits were purchased from RiboBio (Guangzhou, China). Cells were seeded in 6-well plates and transfected at about 80% confluence with Atg5/BECN1 siRNAs and their corresponding negative control siRNA by Lipofectamine 3000 (Invitrogen, Carlsbad, CA) according to the manufacturer’s protocol. Cells transfected for 72 h were harvested for immunoblotting analysis.

### Apoptosis assays

After drug treatment, cells were double-stained with Annexin V-phycoerythrin (PE) and 7-amino-actinomycin (7-AAD) (BD Biosciences) at room temperature in the dark as described in the vendor’s protocol, followed by flow cytometry analysis within 1 h (BD Biosciences). We analyzed proportions of apoptotic cells using FlowJo Version 7.6.1 software (FlowJo, Ashland, OR).

### Xenograft experiments

MKN45 cells detached with trypsin/EDTA (Gibco BRL) and SNU5 cells were collected and re-suspended with PBS to a final concentration of 1 × 10^7^ cells/ml. Then, 100 μl cell suspension was inoculated subcutaneously in the right flank of 6-week-old female Balb/c nude mice (Vital River Laboratories, Beijing, China). When tumor volume reached approximately 100–250 mm^3^, mice bearing GC cells were randomly assigned to treatment groups and given daily PBS, Criz (12.5 mg/kg/day, by gavage), and Voli (5 mg/kg/day, by gavage) alone or in combination with HCQ (60 mg/kg/day, intraperitoneally (i.p.)) for 21 days (*n* = 5). Tumor size and body weight were measured every 3 days and tumor volume (*V*) was calculated by formula: *V* = *L* × *W*^2^/2 (*L*, long diameter of the tumor; *W*, short diameter of the tumor). After the final drug administration, mice were killed and tumors were stripped for western blots. All animal experiments were approved by Peking University Cancer Hospital’s Institutional Animal Care and Use Committee and complied with the internationally recognized Animal Research: Reporting of in vivo Experiments guideline.

### Statistical analysis

All data were representative of three independent experiments and analyzed by GraphPad Prism 5 software. Continuous variables in line with normal distribution were expressed as means ± SD and compared using two-tailed Student’s *t*-test, one-way or two-way analysis of variance (ANOVA) with post hoc Bonferroni correction; otherwise, data were expressed as median with interquartile range and compared using nonparametric Mann–Whitney *U*-test. *P* < 0.05 was considered statistically significant.

## Supplementary information

Figure S1

FigureS2

supplemental material Figure legend
