# Supplementary material for: Targeting autophagy potentiates antitumor activity of Met-TKIs against Met-amplified gastric cancer
Source: Cell Death Dis. 2019 Feb 13;10(2):139. doi: 10.1038/s41419-019-1314-x (PMC6374362; doi:10.1038/s41419-019-1314-x)
Supplement: Supplementary file 3 — supplemental material Figure legend [file 41419_2019_1314_MOESM3_ESM.docx]

**Supplementary material 1:** **Fig. S1.** Impacts of Met-TKIs on Met non-amplified NCI-N87 cells. **a** Cell viability was evaluated by CCK-8 assay in GC cells treated as indicated. **b** NCI-N87 cells were treated with PHA (800nM), SU (4μM), Criz (800nM) or Voli (80nM) for 36 h. Cell lysates were immunoblotted for corresponding proteins.

**Supplementary material 2: Fig. S2.** Impacts of Met-TKIs combined with autophagy inhibition on NBR1 levels in MKN45 cells. Immunoblots for NBR1 were performed in MKN45 cells exposed to Met-TKIs (PHA 200nM, SU 1μM, Criz 100nM or Voli 10nM) with/without autophagy inhibitors (Baf A1 10nM or HCQ 10μM) for 36 h.
